# Multifaceted WNT Signaling at the Crossroads Between Epithelial-Mesenchymal Transition and Autophagy in Glioblastoma

**DOI:** 10.3389/fonc.2020.597743

**Published:** 2020-11-12

**Authors:** Bárbara Paranhos Coelho, Camila Felix de Lima Fernandes, Jacqueline Marcia Boccacino, Maria Clara da Silva Souza, Maria Isabel Melo-Escobar, Rodrigo Nunes Alves, Mariana Brandão Prado, Rebeca Piatniczka Iglesia, Giovanni Cangiano, Giulia La Rocca Mazzaro, Marilene Hohmuth Lopes

**Affiliations:** Laboratory of Neurobiology and Stem Cells, Institute of Biomedical Sciences, Department of Cell and Developmental Biology, University of São Paulo, São Paulo, Brazil

**Keywords:** glioblastoma, autophagy, microautophagy, chaperone-mediated autophagy, epithelial-mesenchymal transition, metabolic reprograming, WNT signaling

## Abstract

Tumor cells can employ epithelial-mesenchymal transition (EMT) or autophagy in reaction to microenvironmental stress. Importantly, EMT and autophagy negatively regulate each other, are able to interconvert, and both have been shown to contribute to drug-resistance in glioblastoma (GBM). EMT has been considered one of the mechanisms that confer invasive properties to GBM cells. Autophagy, on the other hand, may show dual roles as either a GBM-promoter or GBM-suppressor, depending on microenvironmental cues. The Wingless (WNT) signaling pathway regulates a plethora of developmental and biological processes such as cellular proliferation, adhesion and motility. As such, GBM demonstrates deregulation of WNT signaling in favor of tumor initiation, proliferation and invasion. In EMT, WNT signaling promotes induction and stabilization of different EMT activators. WNT activity also represses autophagy, while nutrient deprivation induces β-catenin degradation *via* autophagic machinery. Due to the importance of the WNT pathway to GBM, and the role of WNT signaling in EMT and autophagy, in this review we highlight the effects of the WNT signaling in the regulation of both processes in GBM, and discuss how the crosstalk between EMT and autophagy may ultimately affect tumor biology.

## Introduction

Autophagy and epithelial-mesenchymal transition (EMT) are cellular processes that present an intricate correlation, being associated with the development and progression of different tumors. Particularly, autophagy is an essential process for cell survival in healthy tissues and in several types of cancer. Autophagy allows tumor cells to obtain energy from their own cellular structures, consequently turning cell components into possible energy and nutrient reserves, preserving metabolic rates (1–3). It directly contributes to resistance to stress in the tumor microenvironment that is associated with hypoxia, nutritional deficit, acidosis or genotoxicity (3–6). Additionally, autophagy is able to interconvert with EMT, that, in turn, is a cellular process in which the epithelial cells lose their cell-cell junction proteins, apical-basal polarity, and interaction with the basement membrane to acquire mesenchymal cell characteristics. This leads to functional changes and increased migratory capacity with invasive properties (7, 8). The crosstalk between autophagy and EMT, as well as all different molecular pathways involved in these processes, is of great interest in physiological and pathological contexts, especially in cancer.

In tumor cells, EMT is activated in metastasis and invasion, which allows cells to detach from the basement membrane and connect with other cells, extravasating from its initial location and invading adjacent tissues (6, 9). However, in some types of cancer, such as glioblastoma (GBM), EMT appears to be favored by downregulation of autophagic processes (10), and a better comprehension of this entangled regulation might help to clarify GBM biology.

GBM is the most common and malignant glioma, classified as a grade IV brain tumor derived from glial cells (11). Besides high mitotic activity, microvascular proliferation, necrosis, cellular polymorphism, and substantial infiltrative capacity, this tumor is highly heterogeneous and is mainly characterized by resistance to treatment (12, 13). The standard treatment for newly diagnosed tumors is surgical resection with radiation, followed by chemotherapy with temozolomide (TMZ) (13). Along with radiotherapy and TMZ, bevacizumab, a monoclonal antibody against vascular endothelial growth factor (VEGF), and lomustine, an alkylating agent, are current options for recurrent GBM (14). However, a high recurrence rate and failure to respond to therapy lead patients to an average survival time of 15 months (15, 16). A subpopulation of GBM cells with stem-like features termed glioblastoma stem cells (GSCs) is characterized by self-renewal and differentiation into distinct neural cell types, which greatly contributes to the intratumor heterogeneity of GBM (17). GSCs are associated with GBM maintenance, progression and resistance to therapy, contributing to the highly aggressive phenotype (18, 19).

Several signaling pathways upregulated in GBM are involved in cell survival, growth and invasiveness that sustain tumor development and confer resistance to therapy and to harsh microenvironments (20). Among these pathways, current literature suggests that aberrant activation of the Wingless (WNT) signaling contributes to GBM pathology through different cell processes, such as proliferation (21, 22), motility (23–25), cell fate specification (26), and maintenance of stemness properties (27, 28). Interestingly, WNT signaling pathway emerged as a pivotal player to mediate the crosstalk between autophagy and EMT, regulating molecules and connecting to other important signal transduction cascades, such as the mTOR signaling pathway.

Mammalian target of rapamycin (mTOR) signaling pathway is also upregulated in GBM (29). mTOR can establish two distinct multiprotein complexes: mTORC1, that promotes its effects downstream of PI3K/AKT signaling, regulating a diverse variety of cellular processes such as glucose, lipid and nucleotide metabolism, protein biosynthesis and degradation (30); and mTORC2, responsible for phosphorylation of AKT on Ser473, which stimulates its maximal activation (31). In GBM, the PTEN/PI3K/AKT/mTOR pathway is one of the most deregulated, contributing to tumor development and progression (32). Furthermore, mTOR is highly associated with WNT signaling (33), and both WNT pathway and mTOR complexes are involved with autophagy and EMT in GBM, as will be discussed throughout this review.

Thus, we analyze the current knowledge on the role of WNT signaling in autophagy and EMT in the context of GBM, discussing the existent crosstalk between these processes. Notably, we consider how autophagy, EMT and WNT signaling may be interconnected to promote GBM development, progression and therapy resistance.

## WNT Signaling Pathway and Its Role in Glioblastoma

WNTs are secreted glycoproteins rich in cysteine ​​and composed of 300–400 amino acids (34). WNT signaling is classified as the canonical or β-catenin-dependent pathway, and the non-canonical or β-catenin-independent pathway.

**Figure 1:**
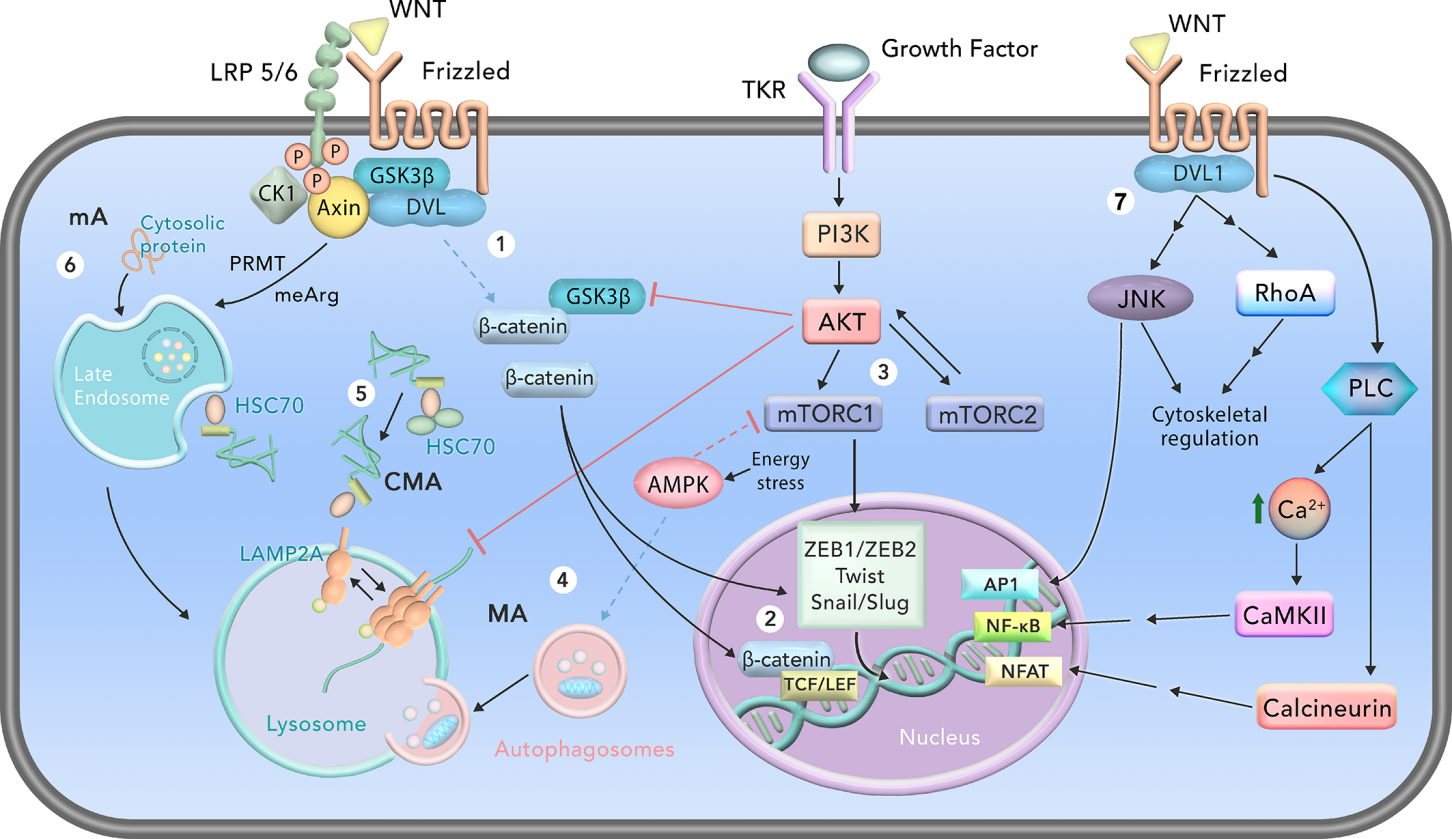
Schematic representation of cell signaling pathways involved in autophagy and epithelial-mesenchymal-transition (EMT). 1) In the canonical WNT signaling, β-catenin stabilizes and translocates to the nucleus after binding of WNT to Frizzled and low-density lipoprotein receptor-related protein 5/6 (LRP5/6) receptors. GSK3 is sequestered together with proteins of the destruction complex of β-catenin (DVL, axin, CK1). 2) In the nucleus, β-catenin binds to TCF/LEF transcriptional factors, activating its target genes. β-catenin also stimulates transcription of important EMT transcriptional factors such as ZEB1/ZEB2, Twist and Snail/Slug. 3) EMT is also induced by the mammalian Target of Rapamycin Complex 1 (mTORC1) signaling, through activation of the transcriptional factors ZEB1/ZEB2, Twist and Snail/Slug. mTORC1 and mTORC2 are activated by tyrosine kinase receptor (TKR) signaling through PI3K/AKT. mTORC2 also stimulates AKT. 4) Depending on the energy status of the cell, activation of AMP Kinase (AMPK) inhibits mTORC1 and induces macroautophagy (MA). 5) Chaperone-mediated autophagy (CMA) degrades cytosolic proteins that possess the KFERQ or KFERQ-like motif through recognition and binding of the heat shock-cognate chaperone of 71 kDa (HSC70) and a cochaperone complex. HSC70 targets the substrate protein to the lysosomal membrane, where it binds monomeric lysosome-associated membrane protein type 2A (LAMP2A) and induces its multimerization and stabilization. The substrate protein is unfolded and translocated into the lysosome for degradation through multimerized LAMP2A. 6) In mammals, microautophagy (mA) occurs in the late endosome, in a process called endosomal microautophagy. It can degrade cytosolic proteins, and some are recognized by HSC70. Some proteins, such as GSK3, are targeted for mA through arginine methylation (meArg) by protein arginine methyltransferases (PRMTs). After entering the late endosome, this organelle fuses with lysosomes to complete the mA cycle. 7) In the noncanonical WNT signaling, WNTs bind to Frizzled receptors and DVL1 is recruited to the membrane. c-Jun N-terminal kinases (JNKs), RhoA and phosphoinositide phospholipase C (PLC) can be activated by noncanonical WNT. JNK and RhoA further regulate the cytoskeleton, and JNK induces gene transcription through activator protein 1 (AP1). PLC increases cytosolic Ca^2+^ levels, leading to activation of Ca^2+^/calmodulin-dependent protein kinase II (CAM-KII) and calcineurin, which activate nuclear factor kappa-light-chain-enhancer of activated B cells (NF-κB) and nuclear factor of activated T cells (NFAT), respectively. Solid black lines represent activation, while solid red lines represent inhibition. Double black lines represent indirect activation. Dashed lines indicate protein interactions when the WNT/β-catenin and mTOR signaling are not activated.

In the canonical WNT signaling (**Figure 1**), WNT proteins (WNT1, WNT3A, WNT7A) bind to the membrane receptor complex of Frizzled (FZD) and low-density lipoprotein receptor-related protein 5/6 (LRP5/6), recruiting Dishevelled (DVL). This ultimately leads to stabilization and translocation of the transcription factor β-catenin to the nucleus, where it activates WNT target genes, such as *AXIN2*, *C-MYC* and *CCND1* (cyclin D1), through binding with the complementary transcription factors T-cell factor (TCF) and the lymphoid enhancer factor (LEF) (**Figure 1**) (34). In the lack of WNT binding, β-catenin is phosphorylated by casein kinase 1 (CK1) and glycogen synthase kinase 3 (GSK3), which leads to the rapid degradation of β-catenin by the proteasome through a destruction complex with involvement of axin-1 and adenomatous polyposis coli (APC) (34). The existence of a low concentration of β-catenin in the nucleus induces the formation of the transcriptional co-repressor Groucho-TCF/LEF complex, which downregulates the expression of WNT target genes (35).

The non-canonical pathway is further divided into two processes: the planar cell polarity (PCP) and the WNT/Ca^2+^ cascade pathways (**Figure 1**). In the first, activation of the PCP pathway leads to determination of the polarity of cells, in addition to affecting cellular shape and migration. In the PCP pathway, WNT ligands (WNT4, WNT5A and WNT11) bind to the FZD receptor and to one of two co-receptors: receptor-like tyrosine kinase (RYK) or receptor tyrosine kinase-like orphan receptor (ROR). This binding induces DVL, which activates DVL-associated activator of morphogenesis 1 (DAAM1), profilin, and the protein Rho- and RAS-related C3 botulinum toxin substrate 1 (RAC1). DAAM1 then activates the RAS homolog family member A (RhoA), which regulates the cytoskeleton through the Rho-associated kinase (ROCK). RAC1 induces c-Jun terminal kinases (JNKs), which directly or indirectly activate cytoskeletal alterations (20, 35). Secondly, the WNT/Ca^2+^ pathway is important during embryogenesis, in the formation of the embryonal dorsal axis, gastrulation and tissue morphogenesis. WNT ligands bind to the FZD receptor, which promotes activation of DVL1 and a G-protein. This complex results in the intracellular release of Ca^2+^, which activates the Ca^2+^/calmodulin-dependent protein kinase II (CAM-KII) and calcineurin, along with two transcription factors: nuclear factor kappa-light-chain-enhancer of activated B cells (NF-κB) and nuclear factor of activated T cells (NFAT). Additionally, certain WNT ligands of the non-canonical pathways can inhibit the canonical WNT pathway (20).

Moreover, certain extracellular antagonists can control WNT signaling, by extracellularly interacting with WNT ligands or receptors, preventing the proper binding of WNT proteins and impairing the maturation of receptors (36). For instance, Dickkopf (DKK1, DKK2, DKK3, DKK4, and DKKL1) is a family of secreted glycoproteins that modulate WNT signaling mainly by binding to LRP5/6, preventing the triggering of signaling (37). These features of DKK proteins are being explored due to its role in different types of cancers (38, 39). Additionally, other antagonists such as WNT inhibitor factor 1 (WIF1), frizzled-related protein family (FRP) – also referred as secreted frizzled-related proteins (sFRP) – and Cerberus work in similar ways (40).

Growing evidence has established a critical role of WNT signaling pivotal players during the onset of GBM. Several of the aforementioned molecules can be associated with the aberrant activation of WNT pathway in GBM, and a particular interest has been attributed to GSCs. WNT5A is epigenetically activated in GSCs due to absence of the H3K27me3 repressive mark (41). Another important molecule is LEF1, and its downregulation inhibits the capacity of self-renewal and expression of stemness markers, such as CD133 and Nestin (42), which negatively impacts proliferation, migration and *in vitro* invasion of GBM cells, playing a role in the EMT process (43). Additionally, the RYK receptor is upregulated in GSCs of patient samples, activating WNT pathway, promoting stemness and improving cell motility (44). Moreover, one of the molecules responsible for translocating and stabilizing β-catenin in GBM is forkhead box protein M1 (FOXM1), which may induce the canonical pathway independently from ligand binding (45). This factor can also be linked to the SOX2 promoter, a classic marker of the stem cell phenotype, known for promoting clonogenic growth in GBM (46).

Using a whole-genome approach, Foltz et al. (47) found that DKK1, sFRP1 and WIF1 were epigenetically silenced in GBM cells (47). The FRP genes are frequently found hypermethylated and inhibited during tumor development. Demethylation of the FRP gene promoter in human glioma cell lines led to an increase in phosphorylated β-catenin in the cytosol, attenuating tumorigenesis (48). Expression of FRPs promotes apoptosis through a possible activation of the DNA damage machinery through FAS-p53, activating the non-canonical WNT/Ca^2+^ pathway and the release of reactive oxygen species (ROS) (49). FRP4 treatment, in conjunction with TMZ, inhibited the canonical WNT pathway and was associated with a decrease in the expression of mesenchymal markers such as N-cadherin, Twist and Snail, along with a greater expression of epithelial markers, such as E-cadherin, showing the role of the inhibitor in reversing EMT (49). In addition, FRP4 chemically sensitizes GSCs, which decreases stemness properties that contribute to therapeutic resistance (49).

WIF1 has a negative influence on the ability of tumor cells to invade and migrate *in vitro* and *in vivo* (24). This suppressor phenotype is due to the downregulation of the canonical and non-canonical WNT pathways, with the inhibition of β-catenin-independent pathway being mediated by the sequestration of WNT5A, a ligand overexpressed in GBM. This inhibition results in decreased phosphorylation of p38-MAPK, reduction of intracellular Ca^2+^ concentration, and reduction in the expression of the metastasis-associated lung adenocarcinoma transcript 1 (MALAT1), a long non-coding RNA and a key invasion regulator (24). Moreover, recovering the expression of DKK1 in GBM cells results in inhibition of the WNT pathway, leading to growth suppression and decreased colony formation (47).

WNT signaling is also fundamental in the crosstalk between microglia and GBM (50). WNT3A derived from GBM induces upregulation of stress-inducible protein 1 (STI1/HOP), interleukin 10 (IL-10), and arginase-1 (ARG-1) in tumor-associated microglia. Together, these molecules collaborate with tumor immune evasion and increase in cell proliferation and migration (51–53). Another report showed that WNT3A increases the level of expression of programmed death-ligand 1 (PD-L1) on the surface of GBM cells, which is an important protein that inhibits the activation, expansion and effector functions of cells with antitumor activity (T^CD8+^) (54), corroborating the importance of WNT3A in immune evasion (55). Furthermore, WNT3A also acts on the WNT/β-catenin/FRA1 axis, a cascade that plays a crucial role in the aggressiveness of glioma and in EMT activation (56).

Due to GBM heterogeneity, EMT is usually incomplete, with associated transient phenotypic states, which results in a combination of epithelial and mesenchymal characteristics (9). It is noteworthy that the infiltrative peripheral region of GBM contains cells with mesenchymal characteristics, with higher WNT/β-catenin pathway activation (5), conferring a specific type of EMT-like process in GBM. As will be further discussed in this review, EMT and autophagy are influenced by WNT signaling, and the crosstalk between these pathways plays an important role in GBM pathology.

## Autophagy in the Context of Glioblastoma and WNT Signaling

Autophagy is a greatly conserved process in eukaryotes, and it has an important role in homeostasis and cell components renewal in the face of adverse conditions. Autophagy degrades defectively folded proteins and dysfunctional organelles, such as mitochondria and peroxisomes, in addition to being essential for cell survival under microenvironmental stress (57).

Mammalian autophagy is classified in three types: microautophagy (mA), chaperone-mediated autophagy (CMA), and macroautophagy (MA) (57, 58). All three types can coexist within a cell and, despite all having the same overall purpose of cargo recycling or degradation of cargo in the lysosome, each one has its own delivery mechanisms and regulation. Remarkably, the modulation of different autophagy types has been associated to GBM biology and will be discussed as follows.

### Microautophagy in Glioblastoma

In mammals, mA ensues through different mechanisms that share some similarities. It can occur directly in lysosomes, in which they extend and wrap themselves around a portion of the cytoplasm, or they form arm- or flap-like protrusions that surround parts of the cytoplasm (59, 60). However, mammalian mA is mostly described as endosomal microautophagy (eMI), occurring in late endosomes/multivesicular bodies (MVBs) (61), which then fuse with lysosomes for breakdown of their cargo (**Figure 1**). This process is attributed to bulk degradation of proteins and other cytosolic components engulfed by late endosomes and can also selectively degrade specific proteins with the assistance of chaperones such as heat shock cognate 71 kDa (HSC70) (61). Interestingly, Sato et al. (62) demonstrated that rapamycin induces eMI, independently of MA, while lacking effect upon CMA (62). This activation is triggered by the nuclear transcription factor EB (TFEB) which increases the expression of several genes related to mA (62).

Recent studies have shown a connection between the WNT pathway and mA. WNT activation leads to the sequestration of GSK3 from the cytosol into late endosomes/MVBs through mA (63). WNT signaling also stabilizes other proteins and GSK3 substrates besides β-catenin. Moreover, WNT activation is highest in the G2/M phase of the cell cycle and the stabilization of GSK3 substrates is thought to decrease the rate of GSK3-dependent protein degradation to prepare for cell division. This shifts the molecules from proteasome degradation (which consumes a large amount of ATP) to microautophagic degradation, which depends on hydrolases inside lysosomes and does not require ATP consumption (64). Despite the research gathered about WNT and mA, there is still an extensive gap in the knowledge of the intricate regulatory mechanisms and the physiological function attributed to mA/eMI in mammals.

eMI is suggested to control protein quality (65) by limiting the intracellular levels of key proteins, working as a regulator of specific cellular processes (66, 67). Other than its physiological roles, mA may be involved with cancer growth. Albrecht et al. (64) showed, for example, that arginine methylation (meArg), promoted by protein arginine methyltransferases (PRMTs), is important for mA, being necessary for the WNT-induced GSK3 sequestration and subsequent microautophagic degradation (64). GSK3 and the tumor suppressor SMAD4 are methylated and targeted to late endosomes/MVBs upon WNT signaling (**Figure 1**), suggesting that inhibition of PRMT1 might oppose cancer progression. Additionally, axin has also been shown as a PRMT1 substrate. As mentioned above, axin acts as a scaffold protein and plays a major role in the destruction complex of β-catenin (64). Upon WNT activation, axin is recruited to the plasma membrane, where it binds to the WNT receptor complex. Arginine methylation in axin has been shown to increase its binding to GSK3, and the WNT coreceptors and GSK3 are then translocated together with axin to MVBs (64).

Thus, meArg has been associated with cancer progression and PRMTs have been considered as novel drug targets (68). In GBM, PRMT2 was shown to be overexpressed and is correlated with a poor prognosis (69). PRMT5 is also highly expressed in GBM, promotes self-renewal of GBM neurospheres (70), and resistance to mTOR inhibition in GBM cells lines and short-term patient cultures (71). mA, however, has not been investigated in GBM. Due to the recent discoveries on the requirement of meArg and mA to WNT signaling activation, this autophagic process might be involved with GBM biology and merits further investigation.

### Chaperone-Mediated Autophagy in Glioblastoma

CMA contributes to the control of cellular quality and maintenance of the proteome (72, 73). Exposure of the KFERQ-like domain of proteins targets them to CMA and selective eIM. In CMA, exposure of this motif leads to its recognition by the chaperone HSC70, and consequent binding of this chaperone to the substrate protein, followed by targeting of this complex directly to the lysosomal membrane (74). This mechanism differs from mA and MA, in which the cargo is sequestered into vesicles before being directed to the lysosome (75). Interestingly, selective blockage of CMA induces activation of MA; however, MA cannot compensate for CMA functions under specific stress conditions, in which CMA plays an essential role (76).

Upon binding of HSC70 to the substrate protein, a chaperone-cochaperone complex is formed and assists HSC70 to redirect client proteins captured in the cytosol to the lysosome membrane (**Figure 1**) (77). On the lysosome membrane, the chaperone complex and its client proteins bind to the lysosome-associated membrane protein type 2A (LAMP2A), inducing LAMP2A multimerization (78). After unfolding, the substrate is then translocated into the lysosome, where the protein is rapidly degraded by hydrolases (77, 78). It is clear that CMA activity is limited by the levels of LAMP2A on the lysosomal membrane, which makes LAMP2A the rate-limiting component for CMA (79, 80).

In addition to LAMP2A levels, further regulation of CMA occurs at the lysosomal membrane. Glial fibrillary acidic protein (GFAP) and elongation factor 1α (EF1α) regulate the stability of multimeric LAMP2A (81). Lysosomal GFAP, when phosphorylated by AKT1 activated by mTORC2, remains bound to EF1α and it is unable to stabilize multimeric LAMP2A (81). Ultimately, this leads to CMA inhibition regulated by mTORC2. When CMA is necessary, the GTPase RAC1 recruits and stabilizes the phosphatase pleckstrin homology domain and leucine-rich repeat protein phosphatase 1 (PHLPP1) at the lysosomal membrane, and this phosphatase dephosphorylates AKT1. This increases non-phosphorylated GFAP and favors the formation of the LAMP2A multimeric complex (73). Thus, it is possible to hypothesize that CMA inhibition might be regulated by mTORC2 and by WNT signaling, since RAC1 is one of the effectors of the PCP pathway. Moreover, signaling of the retinoic acid receptor α (RARα), associated to differentiation of non-tumor and tumor cells, inhibits transcription of genes required for CMA, highlighting an additional pathway of regulation for this autophagic process in health and, particularly, in cancers such as GBM, in which GSCs are a prominent factor (82).

CMA activity is markedly increased in cancer cells. Noteworthy, an anti- or pro-cancer function of CMA appears to depend, at least in some extent, on the status of cell transformation (83) and stage of the specific tumor (84, 85). This facet supports the importance of a context-dependent therapy investigation, requiring specialized research and a more translational approach.

There are very few studies that specifically explore the function of CMA in GBM, but the aggressiveness and high heterogeneity of this tumor raise important questions about the extent to which CMA may be contributing to these features. Maititi et al. (86) reported that the treatment of different GBM cell lines with curcumin and solid lipid curcumin particles downregulated LAMP2A levels, consequently decreasing CMA (86). Alternatively, regarding CMA activation, TMZ-resistant and TMZ-sensitive cells respond differently, according to hypoxia-inducible factor 1α (HIF-1α) expression and their basal levels of cytoplasmic ROS. CMA was shown to be the main process through which TMZ inhibits activity of HIF-1α in sensitive cells, promoting apoptosis and increasing responsiveness to the drug (87). Downregulation of LAMP2A blocks HIF-1α degradation through CMA and is sufficient to induce a TMZ-resistant phenotype in GBM sensitive cells, reducing the expression of several apoptosis-related genes upon treatment (87). Further research showed CMA is activated following cytoplasmic release of mitochondrial ROS, since concomitant increase in LAMP2A and HSC70 is observed with induction of oxidative stress. In accordance, inhibition of mitochondrial ROS downregulates LAMP2A and HSC70 (88). TMZ-sensitive cells present low basal levels of ROS, which in turn are increased with TMZ treatment, consequently activating CMA and pro-apoptotic genes. In contrast, TMZ-resistant cells have high basal levels of ROS and fail to activate CMA upon TMZ treatment (88). Remarkably, knockout of LAMP2A leads to CMA blockage, which is sufficient to trigger the switch of sensitive to resistant responsiveness (88). In T cells, production of mitochondrial ROS by engagement of the T cell receptor induces nuclear translocation of NFAT, which binds to the *LAMP2* promoter and activates CMA (89). As discussed in section 2, the non-canonical β-catenin-independent WNT/Ca^2+^ cascade activates NFAT (90). Although non-canonical WNT pathways are usually related to embryonic patterning and morphogenesis, their aberrant regulation increases invasiveness in GBM (20). It is interesting to interrogate whether non-canonical WNT signaling through NFAT could be involved with activation of CMA in GBM cells, since ROS can be involved with the activation of both NFAT and CMA.

Another compelling mechanism is the ability of GBM cells to induce CMA in pericytes that surround the tumor, suppressing antitumor response and facilitating tumor progression. Specifically, interaction between GBM cells and pericytes of the peritumoral area increases ROS and subsequently LAMP2A levels, inducing nanotube formation and downregulation of the secretion of vesicles containing cytotoxic molecules (91). This results in promotion of direct interaction between these cells and abrogates inflammatory response (91). Supporting this, high levels of granulocyte-macrophage colony stimulating factor (GMCSF), molecule that promotes immunosuppression, were found in GBM cells after interaction with pericytes (91).

A thought-provoking aspect of CMA contribution to GBM is that chaperones and cochaperones that function in CMA play important roles in GBM biology (92). STI1/HOP, a known component of the CMA translocation complex (77), is upregulated in GBM cells. High levels of both STI1/HOP and its partner cellular prion protein (PrP^C^) are correlated with increased proliferation of GBM cells (93). Moreover, interaction between STI1/HOP-PrP^C^ was found to sustain proliferation of GBM stem-like cells and tumor growth *in vivo*, contributing to its aggressiveness (94). Curiously, a mutant form of PrP^C^ interacts with LAMP2A and HSC70, raising the question whether the non-mutated form of PrP^C^ can also interact with CMA-related proteins (95). Interestingly, WNT3A increases STI1/HOP expression in GBM cells (53), suggesting that WNT signaling could regulate CMA, or that GBM cells take advantage of increased STI1/HOP expression to sustain tumor growth and modulate GSCs. Furthermore, since mA favors WNT signaling through GSK3 degradation, these data demonstrate how WNT signaling appears closely involved with autophagic processes, both by depending on them to be induced and by participating in their indirect activation.

In addition to STI1/HOP, other proteins of the translocation complex are proposed to be related to GBM biology, such as BAG1. BAG1 confers proliferation advantage in GBM cell lines in serum starvation conditions, a CMA-inducing environment, through interaction with BCL-2 (96). Although the mechanisms described for the function of these proteins in the biology of GBM have not yet been directly associated with CMA, it may be an interesting strategy to investigate the contribution of CMA in this context, considering the intricate relationship of its components with the aggressiveness of GBM. Thus, evidence gathered here demonstrate that, despite knowing the main processes and molecules involved in CMA, novel intermediate players that finely tune the mechanism - especially in the context of disorders such as cancer - need to be unraveled to actively consider CMA as a viable, well controlled and safe therapeutic approach.

### Macroautophagy in Glioblastoma

MA involves the formation of an isolated vesicle, termed autophagosome, for transportation of the materials to the lysosome (**Figure 1**) (97). It consists of the following critical steps: initiation, nucleation, maturation and degradation of the cargo, with consequent release of the degradation yields to the cytosol (98). In the initiation step, there is the formation of a kinase complex comprised of Unc-51-like kinase 1 and 2 (ULK1/2), family 200-kDa interacting protein (FIP200), the mammalian ATG13 (mATG13); and ATG101 (99). mTORC1 inhibition under starvation activates ULK1/2, triggers phosphorylation of mATG13 and ULK1/2, and induces ULK-dependent phosphorylation of FIP200 (100). Moreover, studies demonstrate AMP-activated protein kinase (AMPK) binds to the ULK1-mTORC1 complex and induces ULK1-mediated autophagy through inhibition of mTORC1 by phosphorylation of Raptor (**Figure 1**) (101, 102).

In the stage of nucleation, the ULK1 complex targets another protein complex, which includes a class III phosphatidylinositol-3 kinase (VPS34), beclin 1, ATG14L, VPS15, and the autophagy beclin 1 regulator 1 (AMBRA-1) (103, 104). Subsequent to phagophore nucleation, in the maturation phase, there is the formation of two protein conjugations. During one of them, the processing of microtubule associated protein 1 light chain 3 (LC3) by ATG4 occurs to yield LC3-I, which in turn is bound to the target lipid phosphatidylethanolamine to generate processed LC3-II (105). LC3-II is integrated in the phagophore where it interacts with adaptor molecules, such as p62/SQSTM1, to select cargo for degradation and to label the phagophore membrane as autophagic (106). Promptly after autophagosome completion, most of the ATG proteins detach from the vesicle (107) triggering its fusion with the lysosome to form the autolysosome (108). In mammalian cells, autophagosome-lysosome fusion is regulated by LAMP2 and the small GTPase RAB7 (109, 110).

The role of MA in cancer and in GBM has been extensively covered in the literature (for more information, refer to (98, 111). Here we will focus on the involvement of the WNT pathway and MA in GBM.

In GBM cell lines, WNT5A/β-catenin signaling was demonstrated to modulate MA (112). The macroautophagic flux was increased upon impairment of WNT/β-catenin signaling *via* the inhibition of the downstream effector TCF (113). Similarly, extracellular DKK1 triggers MA by disrupting the communication of WNT ligands to their cell surface receptors, thus inhibiting WNT cascades (113).

Furthermore, WNT signaling proteins are also targets of MA. The WNT/β-catenin target genes *AXIN2* and *CCND1* become downregulated upon starvation-induced MA or mTOR blockage in GBM, although *C-MYC* expression shows no alteration (114). The same autophagy-inducer mechanisms were shown to decrease the expression of the DVL2 protein (115). This was also observed by Colella et al. (114), where MA led to a decrease in the expression of DVL2 and nuclear β-catenin in GBM cells (114). The study also showed that β-catenin was located in nearby plasma membrane regions and engaged with N-cadherin to form structures similar to epithelial-like cell-cell junctions (114).

The protein Von Hippel-Lindau (VHL) was demonstrated to ubiquitylate DVL2, which leads to its macroautophagic degradation (115). Noteworthy, data from the literature demonstrate that VHL also promotes the ubiquitination of the HIF-1α and 2α (HIF-2α) in GBM (116–119). Interestingly, in renal cell carcinoma, HIF-2α was shown to be degraded *via* both proteasome system and macroautophagic pathway in a VHL-dependent manner. Inhibition of MA led to HIF-2α degradation by the proteasome, whereas suppression of the proteasome caused HIF-2α degradation by MA (120). Furthermore, HIF-1α was demonstrated to induce MA in GBM as a mechanism of resistance to antiangiogenic therapy (121). Evidence indicates an interaction between the WNT/β-catenin pathway and HIF proteins in both physiological and pathological conditions. For instance, the crosstalk between WNT signaling and HIF-1α was demonstrated to be involved in maintenance of GSCs stemness, since their interaction transcriptionally regulates the neuronal differentiation of these cells (122).

In summary, the aforementioned studies point out to the importance of the WNT signaling and MA in GBM biology. As discussed, there is an important crosstalk between WNT/β-catenin signaling, MA and hypoxic pathways that warrants further investigation. The relationship between these pathways indicates the importance of metabolic reprogramming in GBM, in a context where nutrient availability may be scarce, and MA may be downregulated through induction of WNT/β-catenin and PI3K/AKT/mTOR signaling.

## Epithelial-Mesenchymal Transition in Glioblastoma

Epithelial cells can undergo EMT, in which cells lose their apical-basolateral polarity as well as cell-cell adhesion structures in order to obtain an enhanced migratory phenotype (123). Upon transition, the cells become able to degrade the basal membrane using metalloproteases and thus migrate from their original site assisted by cytoskeleton rearrangements (124). As described in the name of the process, the transitioning cell shows increased expression of mesenchymal markers as well as loss of epithelial marker and adhesion molecules (125). Initial onset of EMT is dependent on many proteins that participate in the motility cycle, such as Rho GTPases, RAC1, and CDC42 (126), transcription factors from the SNAI family, such as Snail1 and Slug, as well as homeodomain transcription factors such as ZEB1 and ZEB2 (**Figure 1**) (5, 125). Additionally, many of the above-mentioned proteins - such as Snail1, Slug, ZEB1, ZEB2 and Twist - are able to regulate the switch of expression from E-cadherin to N-cadherin, an important hallmark of EMT (127, 128). This biological phenomenon has a well-known role in wound healing and development (129, 130), in addition to cancer invasion (131). Moreover, EMT is divided into 3 distinct subtypes: type 1 EMT is related to morphogenesis and is modulated by signaling pathways such as WNT/β-catenin, which in turn regulates Snail1 expression (132); type 2 EMT is related to wound healing and fibrosis and can be regulated by Slug, epidermal growth factor (EGF) and transforming growth factor β (TGF-β) pathways (130); and type 3 EMT occurs in cancer cells, playing an important role on tumor invasion and metastasis (133), and can be regulated by cytokines secreted in the tumoral niche such as TGF-β and tumor necrosis factor α (TNFα) (5).

As seen during neurodevelopment, mainly during migration of the neural crest, neuroepithelial cells may undergo transition to a more mesenchymal phenotype and, therefore, increased migratory capacity (134, 135). GBM can co-opt this process to assist cell invasion and migration. Importantly, neural tissues lack E-cadherin expression, although this protein is expressed in GSCs and in a rare subgroup of extremely aggressive GBM cells (5, 136). As GBM cells do not intrinsically express E-cadherin, the switch to N-cadherin, that is classic in EMT, is not entirely observed in this tumor (137). Moreover, distinct motility behaviors observed in GBM were associated to N-cadherin distribution in the cell instead of its expression levels (138). Therefore, GBM cells undergo a process similar to EMT, called EMT-like or glia-to-mesenchymal transition (GMT). Such process can be induced through radiation and be modulated by TGF-β-dependent and TGF-β-independent pathways through mitogen-activated protein kinases (ERK1/2) and GSK3, which in turn modulate Snail expression and localization (139). Likewise, neuronal cells also seem to undergo an EMT-like process, relying on Scratch1 and Scratch2 proteins, members of the Snail superfamily, to repress E-cadherin expression (140).

Additionally, the G protein-coupled receptor LGR5 triggers EMT in GSCs through the WNT/β-catenin cascade (141). Moreover, WNT-C59, an antagonist of the pathway, was able to impair EMT in GSCs (141). The transcription-related protein FRA1 was shown to act downstream of WNT3A signaling, promoting EMT in GBM cells and therefore contributing to the aggressive behavior of this tumor (56). Furthermore, the micro-RNA miR-504 suppresses the expression of the *FZD7* gene, which causes the WNT/β-catenin pathway to be downregulated and affects EMT (142). Importantly, the transcription factors LEF1 and HOXA13 have both been shown to promote EMT in GBM *via* WNT signaling (143). Furthermore, the WNT/β-catenin cascade was shown to be activated in GBM cells of the mesenchymal subtype, leading to induction of expression of ZEB1, Twist1 and Slug, along with increased migratory capability of these cells (5). These recent findings support the importance of the WNT signaling for induction of EMT in GBM, pointing out to the WNT pathway as a promising therapeutic target for reducing the aggressiveness of this tumor.

## Crosstalk Between Autophagy, Epithelial-Mesenchymal Transition, and WNT Signaling in Glioblastoma

**Figure 2:**
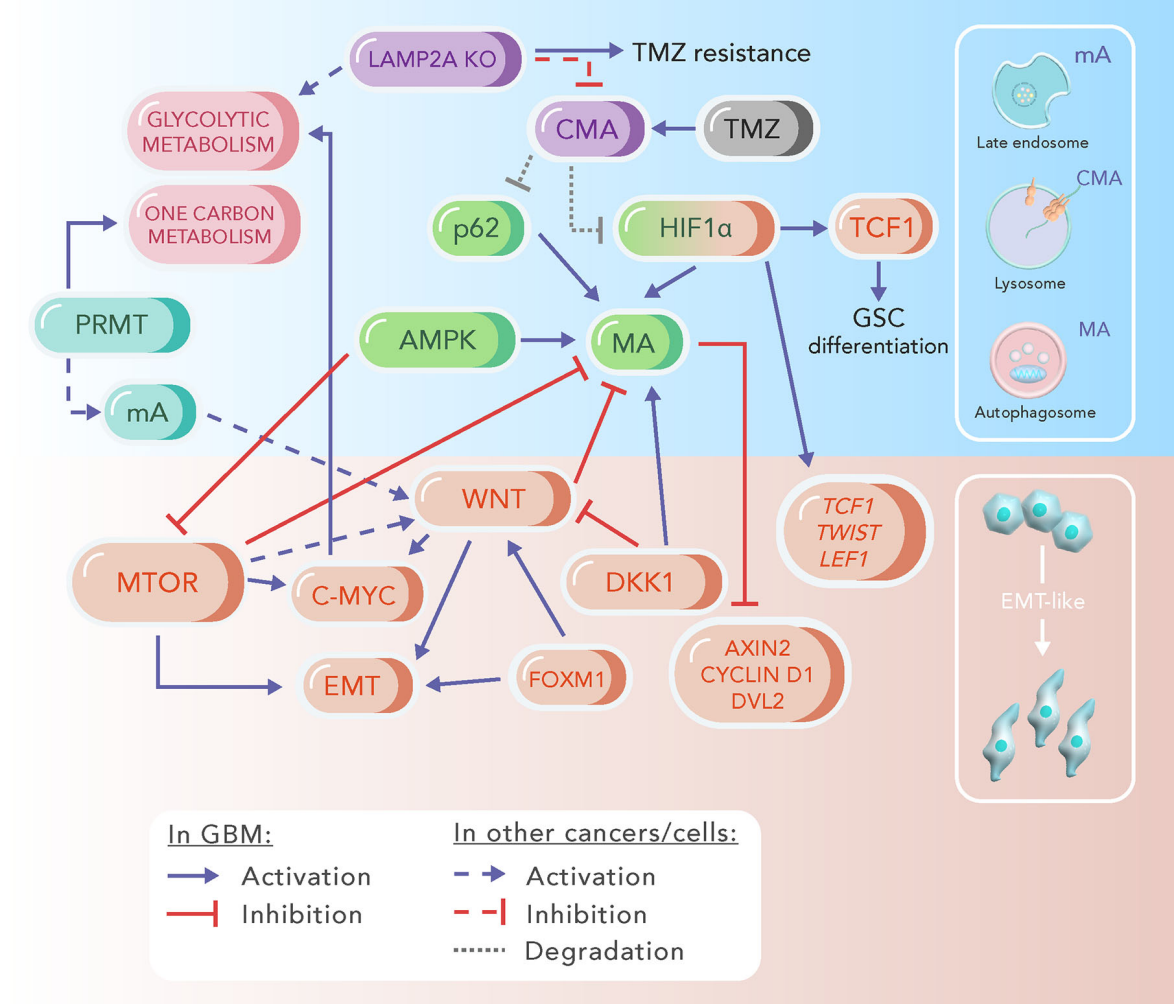
Schematic representation of signaling pathways involved with WNT/β-catenin, autophagy and epithelial-to-mesenchymal transition (EMT) in glioblastoma (GBM). The major players of the highlighted processes are grouped by a colour code, which differentiates microautophagy (mA, light blue), chaperone-mediated autophagy (CMA, purple), macroautophagy (MA, green), EMT (orange) and some metabolic processes (pink). AMPK, AMP Kinase; DKK, Dickkopf; DVL2, dishevelled 2; FOXM1, forkhead box protein M1; HIF1a, hypoxia-inducible factor 1a; LAMP2A KO, knockout of LAMP2A protein; LEF1, lymphoid enhancer factor 1; mTOR, mammalian target of rapamycin; PRMT, protein arginine methyltransferase; TCF1, T-cell factor 1; TMZ, temozolomide.

When supporting cancer cells, autophagy can provide nutrients and energy for both EMT and the consequent metastasis, since it can sustain cells for survival in stressful environmental conditions (7). On the other hand, autophagy can also inhibit EMT by modulating the expression of specific proteins. As such, MA can downregulate mTOR signaling, leading to β-catenin and N-cadherin degradation and E-cadherin expression (7, 144). Therefore, it is believed that autophagy is oncosuppressive at initial stages of metastasis, by destabilizing or degrading crucial inducers of EMT, consequently inhibiting this process. If the cell is unable to prevent malignant transformation and metastasis ensues, EMT is activated and autophagy is required for tumor cell survival in environmental stress (5). Additionally, further dimensions should be added to this hypothesis, by considering the role that important pathways, such as the WNT signaling, and the different autophagic processes (mA, CMA, and MA), may have in the balance of oncosuppressive or metastatic function, especially in a highly infiltrative tumor as GBM. In this session, we discuss the evidences for the crosstalk of WNT signaling-autophagy-EMT observed in different aspects of GBM biology (**Figure 2**).

The microenvironment surrounding GBM presents hypoxic niches, which contribute to scarcity of nutrients and to dependence on glycolytic metabolism (145). Hypoxia, and consequent HIF-1α signaling activation, is a known inducer of MA, and also a known EMT promoter in different types of cancers (146–150). In GBM, an intricate relationship of those pathways with WNT signaling can be postulated, since strong correlation in the expression of HIF-1α and β-catenin was demonstrated in tumor cells (151). In addition, HIF-1α promotes the expression of the β-catenin transcriptional partners TCF1 and LEF1, regulating β-catenin nuclear retention (152, 153). This contributes to the activation of target genes associated with GBM tumor invasion and proliferation, such as Snail/Slug and cyclin D1 (154). Remarkably, MA and CMA appear to have opposed roles during GBM responsiveness to hypoxia. As covered in *Chaperone-Mediated Autophagy in Glioblastoma*, CMA decreases HIF-1α activity in GBM cells. On the other hand, hypoxia induction increases MA. Furthermore, the response to curcumin treatment is also opposed: while the treatment induces MA, concomitantly decreasing the expression of β-catenin targets, it also decreases CMA. However, this increased MA by curcumin treatment could be a feedback response of the tumor cell to the CMA inhibition.

Alternatively, HIF-1α/TCF1 activation can also result in neuronal differentiation in GBM cells subjected to hypoxia, and this pathway is primarily activated in the subpopulation of GSCs, being responsible for a pro-differentiation phenotype (122, 155). Interestingly, HIF-1α/TCF4 activation in normoxia triggers transcriptional inhibition of the same regions once activated in hypoxia (122). The differences between the outcomes for tumor biology might rely on the highly heterogenous cell populations in GBM tumors, and the initial spatial-molecular context of these cells. Despite that, different reports advocate hypoxia-mediated pathways working in tumor supporting mechanisms, contributing to chemotherapy resistance and invasion of healthy tissue.

Besides contributing to the distinct above-mentioned mechanisms, HIF-1α is able to bind the hypoxia-response element of *TWIST* proximal promoter and mediate its expression (156). Twist and Snail are targets of β-catenin signaling (156, 157), and are known EMT transcription factors (158), whose levels can be controlled by MA (159). In GBM particularly, a recent report describes the associated increase in MA markers with decreased levels of Twist and Snail in tumor cells treated with a combination of TMZ and curcumin (160). Moreover, Snail and Twist are found increased in GBM, together with other EMT-related transcripts (161). Thus, regulation of the levels of these transcription factors by autophagy represents one of the feedback correlations contributing to the crosstalk between autophagy and EMT, ultimately dictated by WNT upstream signaling.

An additional factor to be considered when exploring the implications of WNT pathway, EMT and autophagy in GBM is FOXM1. FOXM1 is upregulated in GBM (162) and plays a key role in the EMT-like process, directly controlling the expression of the proteins matrix metallopeptidase 2 (MMP2) (163) and A disintegrin and metalloproteinase 17 (ADAM17) (164). WNT signaling works in the stabilization of FOXM1 (165), and FOXM1 is able to directly translocate β-catenin to the nucleus of GBM cells, independently of extracellular ligands (45). Recently, it was shown that FOXM1 binds to the promoter region and controls the overexpression of the ubiquitin-conjugating enzyme E2C (UBE2C), protecting GBM cells from autophagic cell death, probably through PI3K-AKT-mTOR activation (166).

According with this overall increase in WNT activation in GBM, protein expression of the DVL family is also enhanced in this tumor (167–169). Interestingly, autophagic decrease in DVL2 levels impairs β-catenin nuclear localization, concomitant with an epithelial-like phenotype (114). Additionally, the dishevelled-associated antagonist of β-catenin 2 (DACT2) is a DVL-interacting protein that decreases the level of activated β-catenin resulting in suppression of WNT/β-catenin target genes (170). In GBM, decreased levels of DACT2 were proportionally associated with shorter patient survival, greater tumor aggressiveness and *in vivo* growth (171), suggesting that an apparent decrease in the levels of this protein favors tumor maintenance. The control of the levels of DACT2 in GBM has not yet been directly associated with autophagy, but the expression of other proteins of the same family were found strongly associated with autophagic processes in cancer cells (172, 173). Those evidences support the fundamental role of the intricate relationship between the EMT-like process, WNT signaling and autophagy for the progression of GBM tumors.

In the context of GBM, angiogenesis provides nutrients for the tumor to grow and favors metastasis (174). Thus, antiangiogenic therapy has been employed for GBM, especially with bevacizumab as adjuvant therapy (175). Although bevacizumab was shown to increase progression-free survival by 6 months, its effects on overall survival are controversial (175). Moreover, bevacizumab treatment generated hypoxia in the tumor microenvironment *in vivo*, and this hypoxia induced by the drug altered the metabolism of GBM cells *in vitro* (176). Interestingly, endostatin is another antiangiogenic drug being considered for GBM therapy. Endostatin is a terminal fragment of collagen XVIII-α 1, binds to α5β1 integrin on endothelial cells and induces autophagy through VPS34, beclin 1 and LC3-II activation (177, 178). Moreover, endostatin was shown to antagonize WNT signaling and to target β-catenin for degradation (179). Endostatin also decreased EMT markers on basal cell carcinoma (180). Additionally, a negative correlation between β-catenin and beclin 1 was reported (181), which could explain the decrease of EMT markers by endostatin, through inhibition of WNT signaling and induction of autophagy. Other reports in the literature confirm a role for WNT signaling in inhibiting autophagy in GBM (182).

Therefore, other therapeutic approaches against GBM are aiming to induce autophagy through mTOR inhibition. The first line of mTOR inhibitors, however, were able to block only mTOCR1 activity (183). As seen throughout this review, mTORC2 is involved with GBM invasion and could inhibit CMA. Moreover, during osteoblast differentiation, WNT3A/LRP5 induces mTORC2/AKT signaling downstream of RAC1 (184). To circumvent this ineffectiveness of initial mTOR inhibitors, novel ATP-competitive mTOR kinase inhibitors (TORKIs) were developed to target both mTOR complexes and are being investigated against GBM (5). Torin 1, for example, induced nutrient deprivation and inhibition of mTOR complexes, resulting in β-catenin re-localization and inhibition of migration (114). Moreover, other molecules that induce autophagy in GBM, such as arsenic trioxide, sodium selenite, and cannabinoids, combined with traditional therapy, were able to increase drug-induced cell death (185–187).

In contrast, autophagy inhibition was also proposed as a therapeutic approach against GBM. TMZ was shown to induce autophagy in GBM cells instead of apoptosis. The use of an inhibitor of late stages of autophagy restored TMZ-induced cell death (188). In accordance with this observation, chloroquine and hydroxychloroquine (HCQ) are being considered promising adjuvants in GBM therapy and are currently under phase III clinical trials (5, 189). Up until recently, these drugs were thought to improve median survival on GBM patients through autophagy inhibition. However, the maximum tolerated dose of HCQ was ineffective for autophagy inhibition, demonstrating the drug exerts its antitumoral effects through unknown mechanisms (190). Ultimately, the question of whether activation of autophagy would be beneficial or detrimental as a possible treatment for GBM remains unanswered. Knowledge on the crosstalk of autophagy with other cellular and molecular mechanisms can enlighten the discussion of how induction or suppression of this process should be safely applied in GBM treatment.

The comprehension of the metabolic features of GBM, highly associated with autophagic process, may contribute to circumvent the challenges faced in anti-tumor therapy. In fact, metabolic reprogramming is one of the hallmarks of cancer (191) and, in GBM, it fuels tumor survival, proliferation and invasion (192). Specially for this type of cancer, the upregulated WNT and AKT/mTOR signaling pathways are directly or indirectly involved with regulation of a diverse range of metabolic pathways (192, 193), intricately regulating autophagy processes and EMT.

As discussed in *Macroautophagy in Glioblastoma*, an overall downregulation of mTOR signaling exerted by MA was noted, leading to β-catenin degradation and decrease in WNT signaling (7, 144). On the other hand, mA is important for canonical WNT signaling activation. Evidence suggests methionine is required for the WNT-induced mA through the universal methyl donor S-adenosylmethionine (SAM), which would assist the internalization of GSK3 into late endosomes/MVBs (194). SAM is a known hub metabolite generated through one-carbon metabolism, which involves both folate and methionine cycles (195). Recently, WNT signaling has been emerging as a key regulator of cellular endocytic capacity and studies show endocytosis of extracellular proteins could provide cancer cells with recycle of nutrients required to grow, leading to adaptation and survival in a hostile microenvironment (64). Albrecht et al. (194) demonstrated methionine and SAM are necessary for this WNT-induced endolysosomal activity and extracellular protein degradation (194). In that sense, one-carbon metabolism has been shown to contribute to the *de novo* synthesis of purines and pyrimidines, in addition to nucleotide salvage in several tumors (193). In GBM, the scavenging of hypoxanthine is being considered as a reason for the resistance to anti-folate therapy (193). Furthermore, nearly half of GBM tumors have deleted 5-methylthioadenosine phosphorylase (MTAP), a key enzyme in the methionine salvage pathway (196). This enhances the dependence of the tumor cells for PRMT5 and studies have demonstrated PRMT5 inhibitors show antitumor effects against GBM (197–200). As such, PRMT5 inhibitors are currently being tested in clinical trials in GBM and several solid tumors (NCT02783300).

As one type of autophagic process, CMA has great impact on glucose metabolism (201). Studies with CMA-incompetent mice showed increased glycolysis in hepatocytes, due to decreased degradation of the glycolytic enzymes pyruvate kinase (PK), enolase 1 (eno1) and aldolase A (aldoA) (202). The glycolytic enzymes hexokinase 2 (HK2) and the M2 isoform of pyruvate kinase (PKM2) were also shown to be substrates of CMA degradation, in ovarian and non-small cell lung cancers, respectively (203, 204). GBM significantly boosts glycolysis for energy production through transcriptional and allosteric upregulation of key glycolytic enzymes such as HK2, enolase 2 (eno2) and phosphofructokinase (PFK), while decreasing the entering of pyruvate to the TCA cycle through inhibition of PDH (for more details on GBM metabolism, refer to 193). Interestingly, WNT3A/LRP5 signaling was shown to increase glycolysis during osteoblast differentiation through induction of the glycolytic enzymes GLUT1, HK2, PFK, PDK1 and others (184). Additionally, mTORC2 was shown to influence glycolytic metabolism in GBM through c-MYC activation (205). It would be interesting to evaluate whether canonical WNT signaling could be involved with glycolytic metabolism and CMA regulation in GBM cells, since the canonical WNT pathway is also upregulated in this tumor. Thus, in contrast to the possibility of CMA and MA hindering GBM metabolism and invasiveness, activation of mA may be a strategy employed by this tumor to survive in the surrounding microenvironment.

Given the importance of WNT signaling in GBM biology, the use of WNT pathway inhibitors are being evaluated for GBM therapy [reviewed in (206)]. LGK974 is a promising small-molecule inhibitor that interferes with the palmitoylation of WNTs, an indispensable step for their secretion and consequent binding to receptors. LGK97 was recently shown to have a synergistic effect with TMZ *in vitro*, reducing the clonogenic potential, with decreased expression of CD133, Nestin and SOX2. Importantly, these effects were shown to be independent of *O* ^6^-alkylguanine DNA alkyltransferase (*MGMT*) promoter methylation status (207). Some WNT signaling inhibitors are currently being tested in clinical trials, such as Enzastaurin and alisertib. Enzastaurin inhibits phosphorylation of GSK3 and AKT, through protein kinase Cβ inhibition. However, a phase II trial of Enzastaurin in combination with bevacizumab had response and progression free survival similar to bevacizumab monotherapy, although the drug was well-tolerated (208). Alisertib is an Aurora A kinase (AURKA) inhibitor that decreased WNT signaling in GBM *in vivo* and *in vitro*. AURKA interferes with GSK3/axin/β-catenin through interaction with axin. In patient-derived orthotopic models of GBM resistant to bevacizumab, alisertib prolonged survival, induced mitotic arrest and decreased histone H3 phosphorylation (209). In a recent phase I clinical trial for GBM patients, alisertib was safe and well tolerated (210). Hence, although WNT signaling inhibitors show promising therapeutic rationale and results against GBM, the intricate crosstalk between EMT-autophagy-WNT needs further evaluation to gain a clearer understanding of how these processes and signaling pathways may be used against GBM.

## Concluding Remarks

GBM is a highly heterogeneous and invasive solid tumor and, although there has been certain progress in the search for new therapeutic strategies, treatment of primary and recurrent GBM still remains a great challenge. Several of the signaling and metabolic abnormalities of GBMs are well described, especially those involving WNT, mTOR and EMT (**Figure 2**). However, the contributions of autophagic processes to GBM biology are largely unknown and debated, with clinical trials aiming at apparent opposing objectives. Some focus on autophagy inhibition, while others target mTOR inhibition to stimulate autophagy (211).

Moreover, as a highly heterogeneous tumor, GBM cells in different tumor layers may present distinct autophagic and EMT phenotypes that confer them with the molecular tools necessary to survive, proliferate, and evade therapy. As such, single-cell omics technologies are greatly contributing to the understanding of GBM heterogeneity and identification of subpopulations in the tumor bulk. However, it is crucial to first understand the molecular control and role of autophagy and EMT in GBM, to add to the identification of subpopulations of cells that would be the best target to overcome this disease.

**Figure 3:**
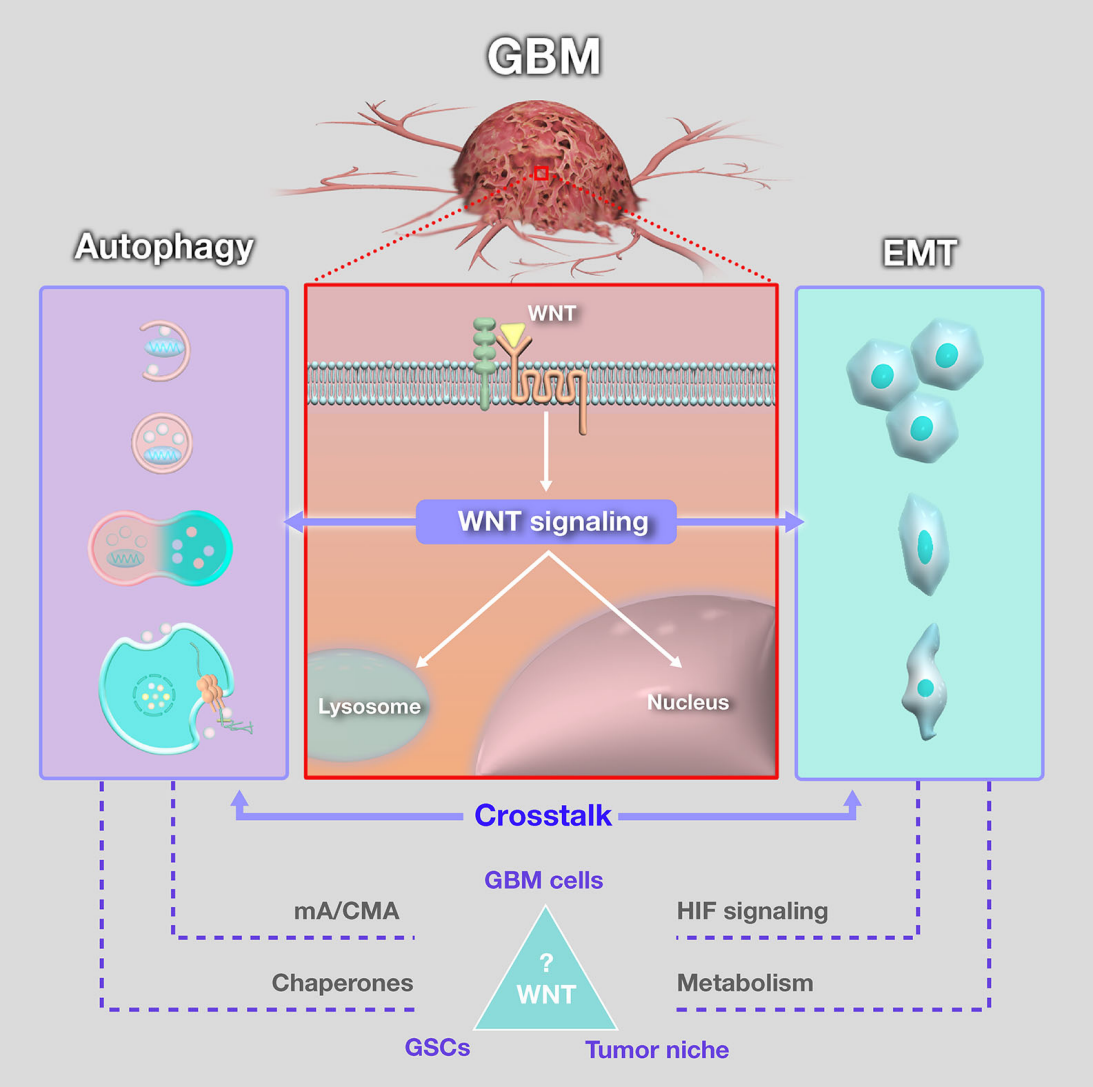
Schematic summary of the crosstalk between WNT signaling, autophagy and epithelial-mesenchymal transition (EMT) in glioblastoma (GBM). Despite the extensively discussed relevance, the importante of WNT pathways in the crosstalk between autophagy and EMT in GBM is still surrounded by open questions. Among them, the role of microautophagy (mA) and chaperone-mediated autophagy (CMA) is largely unknown. Moreover, function of chaperones in this context might represent a target for crosstalk regulation in GBM, in glioblastoma stem-like cells (GSCs), tumor bulk and tumor niche, and warrant further investigation. Accordingly, the role of HIF signaling and metabolic reprogramming on this crosstalk should also be a subject for future research efforts.

As discussed in the present review, the WNT/β-catenin pathway plays a major role on EMT induction and invasiveness in GBM, while contributing to inhibition of MA (**Figure 2**). Interestingly, mA and one carbon metabolism are facilitators of β-catenin activation, and one carbon metabolism has been viewed as a possible therapeutic target in GBM (193). CMA was linked to acquired TMZ resistance in GBM cells, with very few reports about CMA in this tumor. Importantly, CMA assists the metabolic regulation of glycolytic enzymes and lipolysis and can also inhibit MA *via* p62 and HIF-1α degradation (**Figure 2**). Thus, there is an increasing demand for investigating mA, CMA and MA separately from each other in cancer and GBM (**Figure 3**). This could unveil the intricate relationship between these different autophagic processes, and how they influence cancer metabolism, aggressiveness, and invasion. Although these processes have overlapping signaling control, they can be activated or inhibited separately, which could confer tumor cells with a vast array of possible responses to survive and thrive even in harsh microenvironments.

## Author Contributions

Review conceptualization: BPC and MHL. Writing: BPC, CFLF, RPI, RNA, MIME, MBP, MCSS, JMB, GLRM, and GC. Review and editing: BPC, CFLF, RNA, MIME, MBP, MCSS, JMB, and MHL. Post-revision writing: BPC, CFLF, JMB, MCSS, GC, and GLRM. Post-revision editing: BPC, CFLF, and MHL. Figure conceptualization: BPC and MHL. Figure design: MIME. All authors contributed to the article and approved the submitted version.

## Funding

This study was supported by Fundação de Amparo à Pesquisa do Estado de São Paulo (FAPESP, Processes numbers: BPC: 2019/14952-0; CFLF: 2019/14741-9; RPI: 2019/12710-9; MBP: 2017/26158-0; MIME: 2019/11097-1; RNA: 2020/04687-4; MCSS: 2019/06971-4; JMB: 2020/07450-5; GLRM: 2019/14552-1; MHL: 2018/15557-4;) and Conselho Nacional de Desenvolvimento Científico e Tecnológico (CNPq, Process number: JMB: 101796/2020-0).

## Conflict of Interest

The authors declare that the research was conducted in the absence of any commercial or financial relationships that could be construed as a potential conflict of interest.

## Abbreviations

AMPK, AMP Kinase; DKK, Dickkopf; DVL2, dishevelled 2; FOXM1, forkhead box protein M1; HIF1α, hypoxia-inducible factor 1α; LAMP2A KO, knockout of LAMP2A protein; LEF1, lymphoid enhancer factor 1; mTOR, mammalian target of rapamycin; PRMT, protein arginine methyltransferase; TCF1, T-cell factor 1; TMZ, temozolomide.
